# Risk Adjustment in the Medicare Advantage Population Using Encounter Data

**DOI:** 10.1111/1475-6773.70165

**Published:** 2026-09-10

**Authors:** Caroline S. Carlin, Roger Feldman, Jeah Jung

**Affiliations:** ^1^ Department of Family Medicine and Community Health, School of Medicine University of Minnesota Minneapolis Minnesota USA; ^2^ Division of Health Policy and Management, School of Public Health University of Minnesota Minneapolis Minnesota USA; ^3^ Department of Health Administration and Policy, College of Public Health George Mason University Fairfax Virginia USA

**Keywords:** encounter data, medicare advantage, risk adjustment

## Abstract

**Objective:**

To estimate the Centers for Medicare and Medicaid Services (CMS) Hierarchical Condition Category (HCC) risk model using Medicare Advantage (MA) encounter data, as an initial step toward recalibrating risk‐adjusted MA payments.

**Data Sources and Study Setting:**

A 20% sample of Traditional Medicare (TM) claims and MA encounter data for 2016–2022. Standardized fee schedules provide a measure of resource use in TM claims and MA encounters.

**Study Design:**

Ordinary least squares regression replication of the CMS HCC version 28 model for relative resource use among community‐dwelling, non‐dual aged and disabled individuals. Robustness testing includes replication with version 22 model structure, sensitivity to MA chart review records, MA contracts with complete encounter data, and patterns of care during the COVID pandemic.

**Principal Findings:**

Using TM data from 2016 to 2022 results in modest changes in estimated coefficients and 1.7% lower average HCC scores, relative to 2018–2019 TM data used for CMS's HCC model v28. Using MA data result in 8.9% lower average scores than TM‐based scores. The differences between MA‐ and TM‐based HCC scores vary across the distribution of scores. When re‐estimating HCC v28 coefficients, increasing trends in TM and MA diagnosis prevalence are associated with smaller (diluted) coefficients in TM and MA‐based HCC risk models. Trends in medical technology can increase (e.g., high‐cost targeted cancer therapies) or decrease (e.g., lower‐cost biosimilars) HCC model coefficients, with evidence of larger technology‐related decreases in an MA‐based model. The decrease in MA‐based scores does not create new disincentives to enroll beneficiaries who are racial/ethnic minorities or rural residents.

**Conclusions:**

We make an important contribution to the policy debate about MA risk adjustment. Any changes in risk‐adjusted MA payment need to be reviewed in the full context of MA payment policy and MA plan enrollment incentives.

## Callout Box

- What is known on this topic
  ○ Risk adjustment using the Centers for Medicare and Medicaid Services (CMS) Hierarchical Condition Category version 28 (HCC v28) risk model is a critical factor in reimbursement of Medicare Advantage (MA) plans.
  ○ Differences in populations, patterns of care, and coding intensity between Traditional Medicare (TM) and MA have led policymakers to propose recalibrating risk adjustment for MA plans based on MA data.
- What this study found
  ○ When re‐estimating HCC v28 coefficients, increasing trends in TM and MA diagnosis prevalence are associated with smaller (diluted) coefficients in TM‐ and MA‐based HCC risk models.
  ○ Trends in medical technology can increase (e.g., high‐cost targeted cancer therapies) or decrease (e.g., lower‐cost biosimilars) HCC model coefficients, with evidence of larger technology‐related decreases in an MA‐based model.
  ○ MA‐based HCC scores are 8.9% lower than TM‐based scores, without creating additional disincentives to enroll racial/ethnic minority (vs. non‐Hispanic White) or rural (vs. urban) beneficiaries.

## 1 Introduction

Health risk adjustment is the practice of modifying an expected outcome, such as predicted per‐capita expense or a quality measure, to reflect the health risk profile of a group. Risk adjustment has been a feature of contracting with private Medicare Advantage (MA) plans since the original rules were promulgated in 1985 and coverage through private “Medicare Part C” plans began. The Centers for Medicare and Medicaid Services (CMS) Hierarchical Condition Categories (HCC) risk models were developed to incorporate diagnostic information with demographic factors to predict relative spending based on Traditional Medicare (TM) data [1]. The HCC models were phased in, beginning in 2004, to risk‐adjust payments for MA plans [2]. Risk adjustment is a critical part of creating an equitable payment scheme and reducing plans' incentives to seek healthy enrollees to hold down costs.

Policymakers have questioned the equity and efficiency of using a risk model based on TM claims data to adjust MA payments, given differences in the MA and TM environments. Unlike TM providers, MA plans may use provider networks, primary care referrals and prior authorization to manage patterns of care [3]. Because MA plan payment is linked to the measured health risk of enrollees based on clinical diagnoses, plans and their providers have an incentive to document all possible diagnoses present in their population. This incentive for diagnosis coding intensity among TM providers is limited to those participating in the Accountable Care Organization (ACO) program. Differences between MA and TM coding intensity, and the role played by chart review records in the MA encounter data, are well documented [4, 5, 6, 7, 8, 9]. Finally, MA plans attract a mix of beneficiaries that is more racially diverse and less rural than TM beneficiaries [6], factors linked to disparities in access to and use of health care services [10, 11]. For all these reasons, TM claims data may not provide an efficient and equitable basis for risk‐adjusted payment to MA plans. In developing MA‐based risk adjustment, it is important that we check for new disincentives to enroll vulnerable populations.

CMS has expressed its intention to develop MA risk adjusters based on MA encounter data [12]. This concept was explored by a summit of subject matter experts convened by the Urban Institute and the American Action Forum (AAF) in 2018. The complexity of transitioning from the current TM‐based risk adjustment model to an MA‐based model is well documented in their summary [13], with a call for a deliberative, public process of recalibration. Highlighted themes include the importance of balancing payment accuracy with incentives to enroll a broad array of risks, the impact on uncompensated activities such as care coordination, and the ripple effect of MA‐based risk adjustment across the payment mechanism. For example, if a separate MA‐based risk adjustment model were adopted, policymakers would need to debate whether to de‐link MA payments fully from TM data, as summarized in a 2026 MedPAC presentation [14].

Understanding whether and where MA‐based models differ from TM‐based models is a first step in this examination of MA risk adjustment. Expected resource use for certain conditions may differ between MA and TM because MA plans limit services for those conditions, provide better coordination of care, or adopt cost‐saving technologies more quickly [13, 15]. In addition, differences in coding intensity present in TM claims and MA encounter data will impact the model's estimated coefficients. We begin to fill this knowledge gap by identifying influential coefficient changes due to re‐estimation of the model coefficients and examining the impact of coefficient changes on overall risk scores.

## 2 Methods

### 2.1 Study Sample

We used a 20% sample of TM beneficiaries and MA enrollees in the 50 US states or the District of Columbia with 2016–2021 diagnoses and 2017–2022 relative resource use, excluding those with Medicare eligibility due to end‐stage renal disease. This six‐year sample increases sample size and balances recency of data with the need to smooth out COVID‐related distortions with pre‐COVID observations. In keeping with the current HCC algorithm, diagnoses from 2016 to 2021 were used to develop lagged indicators of chronic conditions for beneficiaries with 2017–2022 TM or MA coverage. To be included in a particular year, the individual must have exclusively TM or MA coverage in that year.

The HCC model is estimated for nine enrollee groups spanning community‐dwelling vs. institutionalized individuals, non‐dual vs. dual coverage, aged vs. disabled individuals, and continuing coverage vs. new entrants. We used the two largest of the nine populations—aged, community‐dwelling, non‐dual coverage (CNA) and disabled, community‐dwelling, non‐dual coverage (CND) – for coefficient estimation.

### 2.2 Development of Relative Resource Use

For each MA beneficiary year, we defined resource use from claims and encounters in Carrier, Durable Medical Equipment (DME), Inpatient Facility, Outpatient Facility and Hospice files. The MA encounter data do not include private plans' proprietary payment information. We used the methods detailed by Jung et al. [16] to develop calendar‐year “standardized fee schedules” based on fee schedules and relative values used in TM reimbursement. We also used their methods to clean the encounter data, eliminating duplicate and seemingly erroneous encounters, though without claim processing indicators we are unable to ensure all denied claims have been eliminated. Standardized fees follow the CMS payment structure, including varying professional payments when facility fees are paid, and using Healthcare Common Procedure Coding System (HCPCS) modifiers to adjust payments, such as reductions for bilateral surgeries. With these standardized fees and enrollee months of coverage, we computed resource use per‐member, per‐month (PMPM) for each enrollee year. We divided by the mean resource use PMPM for the full MA sample in that year (all nine populations), to get a measure of relative resource use for that enrollee year based on standardized fees. Within a calendar year, relative resource use averages 1.0 across the nine populations in the MA (or TM) cohort but does not average 1.0 within their CNA or CND subsets.

We applied the same standardized fee schedules and methods to the TM claims data, so we could measure TM relative resource use with standardized fees. As a robustness check, we used TM allowed amounts (actual eligible payer and beneficiary payments) to compute an alternative measure of TM relative resource use.

### 2.3 Development of HCC Model Covariates

We used the HCC software released by CMS [1] in 2025 to compute the demographic and lagged HCC indicators for the presence of the chronic conditions in the model. Our baseline analysis relies on the version 28 (v28) model structure comprising demographic indicators, 115 HCCs, HCC interactions, and a count of conditions identified. Eligible diagnosis codes were obtained from Inpatient facility files and Carrier and Outpatient facility claims that include a valid HCPCS for the HCC risk model [17]. For MA enrollees, we included chart review records (CRR) because CMS currently uses CRR in their risk adjustment. In robustness testing, we isolate the impact of removing CRR and health risk assessment (HRA) records, as well as the impact of restricting the MA sample to contracts that submitted reasonably complete encounter data [16].

A major update to International Classification of Diseases, 10th Revision (ICD10) coding was introduced on October 1, 2022. Because HCC v28 software is not available prior to 2024 [1], we are unable to apply v28 diagnoses mappings that capture codes retired in this update. The change most relevant to our disabled and over‐65 population was a separation of dementia diagnoses by severity level. We manually captured older dementia ICD10 codes to maximize measured dementia prevalence. Because the CNA and CND coefficients are constrained to be equal across dementia severity levels, the use of older codes should not impact our results.

### 2.4 Model Estimation

We estimated the models with ordinary least squares regression. Relative resource use is the dependent variable, and the HCC v28 demographic and HCC‐based indicators are the independent variables. Because our goal was to compare models based on TM vs. MA patterns of care, we merely estimated the coefficients for the existing set of independent variables and did not attempt to validate the appropriateness of the model structure for MA enrollees. In the estimation, we constrained coefficients to be equal across hierarchies where the CMS coefficients are held constant (e.g., across the three dementia severities), as documented in Exhibit A‐1. In addition, because the HCC risk adjustment model is not intended for causal analysis, we did not attempt to control for unobserved factors that could cause selection bias.

We estimated two CNA and CND models in our baseline analysis, summarized in Table 1. The first model (“TM‐based”) was a straightforward replication of the current v28 model using TM data and relative resource use based on standardized fees. A comparison of the replicated coefficients with the current CMS v28 coefficients should capture average differences in patterns of care in a TM population from the CMS biennium of 2018–2019 to our 2016–2022 timeframe. In our second model (“MA‐based”) we estimated coefficients using MA encounter data with relative resource use based on standardized fees to identify how coefficients are impacted by differences in patterns of care and diagnosis coding intensity in the MA vs. TM populations. MA‐based coefficients are also impacted by normalization of the dependent variable—relative resource use—to the overall average MA resource use, rather than the overall average TM resource use.

**TABLE 1:** Summary of risk models.

Model	Estimated by	Data used for model development			HCC scores computed	Color code for graphics
		Sector	Diagnoses	Resource use		
CMS HCC model (version 28)	CMS	TM	2018	2019 allowed amounts	TM population	Gray
					MA population	
TM‐based model	Authors	TM	2016–2021	2017–2022 standardized fees	TM population	Green
					MA population	
MA‐based model	Authors	MA	2016–2021	2017–2022 standardized fees	MA population	Blue

Abbreviations: CMS HCC = Centers for Medicare and Medicaid Services Hierarchical Condition Category; MA = Medicare Advantage; TM = Traditional Medicare.

After estimating the TM‐based model, we used the coefficients to compute TM‐based HCC scores in the TM population. A comparison of scores from the published v28 coefficients to our new TM‐based scores identifies the impact of the change in the time period. We then applied TM‐based and MA‐based coefficients to the MA population to identify how differences in data sources impact risk adjustment in an MA population. In all analyses, we compare HCC score summary statistics (mean, percentiles), demographic coefficients, and the ten most influential HCC coefficients. Predictive ratios by age, sex, and rurality were examined to understand equity impacts in protected populations.

### 2.5 Robustness Testing

In robustness testing, we first estimated the TM‐based model using relative resource use from allowed amounts to see how that difference in resource use measurement impacted HCC scores in a TM population. Second, we estimated the v22 HCC model, in use during our sample period, to assess how recent changes in the HCC model and ICD‐10 mapping impact our comparison of HCC scores and coefficients. Third, we estimated the MA‐based model excluding diagnoses seen exclusively in CRR/HRA, and excluding contracts with incomplete data submission, to identify how the scope of MA data affects the distribution of HCC scores. Finally, we estimated both models excluding years affected by COVID (2020–2022 with lagged diagnoses from 2019 to 2021).

## 3 Results

### 3.1 Descriptive Statistics for the Sample Population

Exhibit A‐2 provides descriptive statistics for the combined CNA and CND populations used to estimate the models. Because of our large sample size, all TM/MA differences are statistically significant. The largest differences include greater racial diversity in the MA population, greater rurality in the TM population, and 5.2% higher health risk paired with 20.7% lower resource use in the MA population. Exhibits A‐3 and A‐4 provide separate statistics for the CNA and CND populations.

### 3.2 TM‐Based Model

**FIGURE 1:**
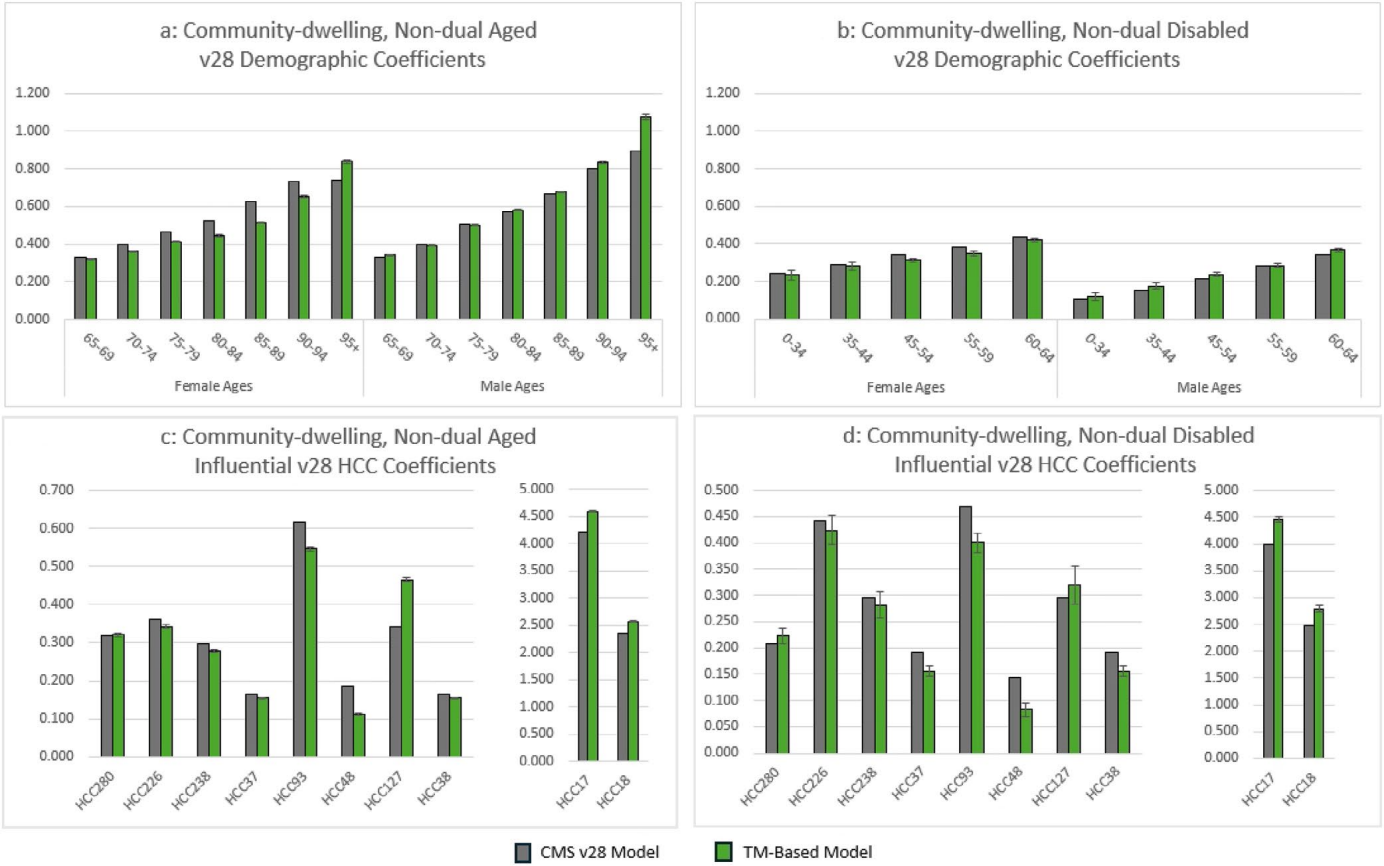
CMS v28 and TM‐based models—demographic and influential HCC coefficients. *Notes:* (1) CMS v28 are published coefficients in the CMS v28 software. (2) TM‐based coefficients are estimated from TM data using standardized fees to compute relative resource use. (3) HCC coefficients that are influential due to high prevalence vs. high acuity are shown separately to allow for differently proportioned *y*‐axes. (4) Influential HCC v28 descriptors in descending order of contribution to mean HCC score, based on CMS published coefficients applied to combined TM/MA and CNA/CND populations: HCC280: Chronic obstructive pulmonary disease, interstitial lung disorders and other chronic lung disorders; HCC226: Heart failure, except end‐stage and acute; HCC238: Specified heart arrhythmias; HCC37: Diabetes with chronic complications; HCC17: Cancer metastatic to lung, liver, brain and other organs and acute myeloid leukemia except promyelocytic; HCC93: Rheumatoid arthritis and other specified rheumatic disorders; HCC48: Morbid obesity; HCC127: Dementia, mild or unspecified; HCC18: Cancer metastatic to bone, other and unspecified metastatic cancer, and acute leukemia except myeloid; HCC38: Diabetes with glycemic, unspecified or no complications. Abbreviations: CMS = Centers for Medicare and Medicaid Services; CNA = community‐dwelling, non‐dual aged population; CND = community‐dwelling, non‐dual disabled population; HCC = Hierarchical Condition Category; MA = Medicare Advantage; TM = Traditional Medicare.

Our first estimation used TM claims data and standardized fees, from which we estimated the CNA and CND coefficients in the TM‐based model. We applied these coefficients to 2022 TM CNA and CND beneficiaries to compute HCC scores. Table 2 compares the sample means and percentiles from the TM‐based HCC scores to the HCC distribution based on CMS v28 coefficients. While the overall difference in means is only a 1.7% decrease (0.99 to 0.97), there are more substantive changes across the distribution of HCC scores, including substantial decreases at the 50th (−9.8%) and 75th (−6.1%) percentiles, with substantial increases in the upper tail (+8.8% at the 99th percentile). To understand the differences in greater detail, we compare individual coefficients in Figure 1. We focus on the primary demographic coefficients and the HCC coefficients for the 10 most “influential” conditions. We identified influential HCCs based on their contributions to the CMS v28 HCC score, averaged across both TM and MA populations. An HCC can be influential through high prevalence or high acuity. On average, demographics contribute 42.5% of the HCC score, and the 10 most influential HCCs contribute 26.6%. The remainder of the score (30.9%) is based on the other 105 HCCs and HCC‐based interactions and condition counts.

**TABLE 2:** Distribution of v28 HCC scores from CMS and TM‐based coefficients, applied to the TM population.

	Using CMS coefficients	Using TM‐based coefficients	Difference
Mean	0.988	0.971	−0.017 [−1.7%]
Percentiles			
1%	0.283	0.284	0.001 [0.4%]
5%	0.330	0.321	−0.009 [−2.6%]
10%	0.332	0.340	0.008 [2.4%]
25%	0.396	0.393	−0.003 [−0.7%]
50%	0.664	0.599	−0.065 [−9.8%]
75%	1.151	1.081	−0.070 [−6.1%]
90%	1.982	1.975	−0.007 [−0.4%]
95%	2.815	2.939	0.124 [4.4%]
99%	5.165	5.620	0.455 [8.8%]

*Note:* CMS v28 distribution based on published coefficients in the CMS v28 software, applied to 2022 TM CNA and CND beneficiaries. TM‐based distribution based on coefficients estimated from TM data using standardized fees to compute relative resource use, applied to 2022 TM CNA and CND beneficiaries.

Abbreviations: CMS = Centers for Medicare and Medicaid Services; CNA = community‐dwelling, non‐dual aged population; CND = community‐dwelling, non‐dual disabled population; HCC = Hierarchical Condition Category; TM = Traditional Medicare.

In panel 1a of Figure 1, when comparing CMS and TM‐based coefficients for females, the aging trend is shallower at most ages in the TM‐based coefficients. For both men and women, the impact of being in the oldest CNA age group is higher in our replicated model than in the published coefficients. In panels 1c and 1d, eight of the most influential HCCs achieved their influential impact through high prevalence, and two through high acuity. While TM‐based HCC coefficients and published coefficients are generally similar, we see large percentage differences for the coefficients of HCC93 (Rheumatoid Arthritis and Other Specified Inflammatory Rheumatic Disorders), HCC48 (Morbid Obesity), HCC127 (Dementia), and HCC17‐HCC18 (both Metastatic Cancers). Because treatment of rheumatoid arthritis (RA) and metastatic cancer is rapidly evolving—biosimilars are dramatically reducing the cost of RA treatment (HCC93), and metastatic cancer (HCC17‐HCC18) therapies are increasingly reliant on high‐cost targeted therapy and immunotherapy—it is not surprising to see coefficients change based on more recent data.

Our data suggest increased coding intensity for morbid obesity (HCC48) in TM populations (Exhibit A‐5), possibly through spillover from providers' MA coding or incentives in TM's ACO program. The 53% increase in prevalence of morbid obesity from TM diagnosis coding far outpaces the 26% growth estimated by Centers for Disease Control over a similar time period (2013–2014 to 2021–2023) [18] and the growth in the MA population (+37%). If TM coding practices for morbid obesity are outpacing growth in true prevalence, we would expect the cost of morbid obesity to include people with marginal diagnoses, diminishing the magnitude of the coefficient.

Differences in the dementia coefficients (HCC127) are harder to interpret. Our manual adjustment to the v28 mapping to capture older dementia codes may be insufficient. If we undercounted dementia prevalence, we may have excluded those with marginal diagnoses from estimation of the coefficient, increasing its magnitude. TM‐based and CMS coefficients for HCC127 in the CND population are much closer. Because dementia may influence disability qualification, we expect fewer marginal diagnoses in this population.

### 3.3 MA‐Based Model

The MA‐based model used standardized fees and MA encounter data to estimate the demographic and HCC coefficients. We applied both the TM‐based and MA‐based coefficients to the 2022 MA population to identify how the estimation data source (TM vs. MA) impacts HCC scores in the MA population.

Table 3 summarizes the distribution of these scores. The MA‐based coefficients reduce the mean HCC score and most percentiles relative to those computed from TM‐based coefficients. In the MA population, mean scores decrease from 1.03 to 0.94 (−8.9%), driven primarily by differences at the median of the distribution and above, and peaking at −12.3% (50th percentile) and −9.9% (75th).

**TABLE 3:** Distribution of HCC scores from TM‐based and MA‐based coefficients, applied to the MA population.

	Using TM‐based coefficients	Using MA‐based coefficients	Difference
Mean	1.033	0.941	−0.092 [−8.9%]
Percentiles			
1%	0.321	0.315	−0.007 [−2.1%]
5%	0.321	0.315	−0.007 [−2.1%]
10%	0.340	0.344	0.004 [1.2%]
25%	0.412	0.398	−0.014 [−3.3%]
50%	0.663	0.590	−0.073 [−11.0%]
75%	1.182	1.037	−0.146 [−12.3%]
90%	2.113	1.904	−0.208 [−9.9%]
95%	3.045	2.771	−0.274 [−9.0%]
99%	5.696	5.419	−0.277 [−4.9%]

*Note:* TM‐Based distribution based on coefficients estimated from TM data using standardized fees to compute relative resource use, applied to 2022 MA CNA and CND beneficiaries. Note this does not match the distribution in Table 2 because the TM‐Based coefficients are applied to the TM population in Table 2, but applied to the MA population in Table 3. MA‐Based distribution based on coefficients estimated from MA data using standardized fees to compute relative resource use, applied to 2022 MA CNA and CND beneficiaries.

Abbreviations: CMS = Centers for Medicare and Medicaid Services; CNA = community‐dwelling, non‐dual aged population; CND = community‐dwelling, non‐dual disabled population; HCC = Hierarchical Condition Category; MA = Medicare Advantage; TM = Traditional Medicare.

**FIGURE 2:**
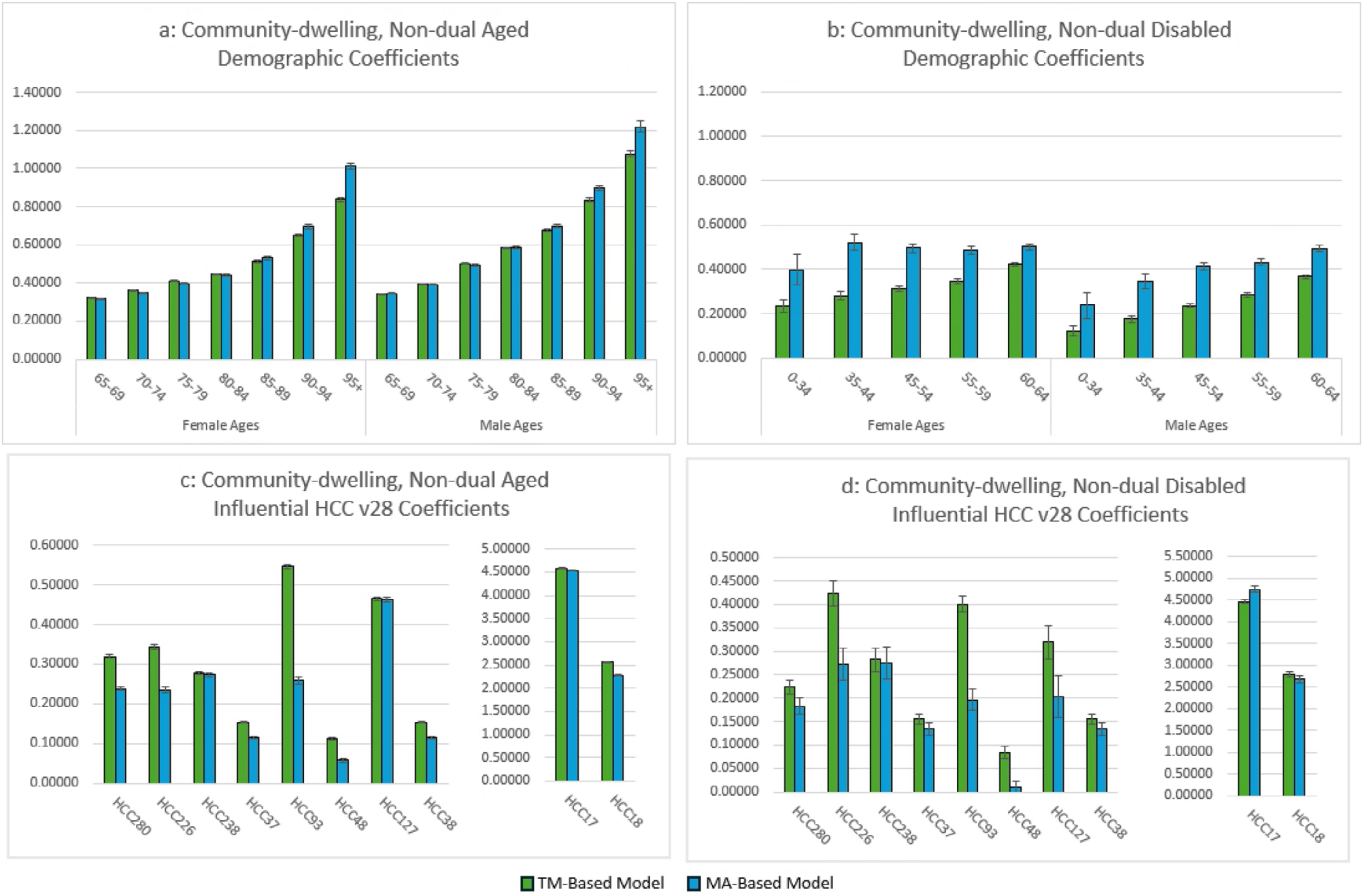
TM‐based and MA‐based models—demographic and influential HCC coefficients. *Notes:* (1) TM‐Based coefficients are estimated from TM data using standardized fees to compute relative resource use and applied to the MA population. (2) MA‐based coefficients are estimated from MA data using standardized fees to compute relative resource use. (3) Influential HCC v28 descriptors in descending order of contribution to mean HCC score, based on CMS published coefficients applied to combined TM/MA and CNA/CND populations: HCC280: Chronic obstructive pulmonary disease, interstitial lung disorders and other chronic lung disorders; HCC226: Heart failure, except end‐stage and acute; HCC238: Specified heart arrhythmias; HCC37: Diabetes with chronic complications; HCC17: Cancer metastatic to lung, liver, brain and other organs and acute myeloid leukemia except promyelocytic; HCC93: Rheumatoid arthritis and other specified rheumatic disorders; HCC48: Morbid obesity; HCC127: Dementia, mild or unspecified; HCC18: Cancer metastatic to bone, other and unspecified metastatic cancer, and acute leukemia except myeloid; HCC38: Diabetes with glycemic, unspecified or no complications. Abbreviations: CNA = community‐dwelling, non‐dual aged population; CND = community‐dwelling, non‐dual disabled population; HCC = Hierarchical Condition Category; MA = Medicare Advantage; TM = Traditional Medicare.

Figure 2 summarizes the demographic and influential HCC coefficients for the MA‐based model, with the TM‐based coefficients shown for reference. The aging slope for CNA (panel 2a) is steeper in the MA‐based model than the TM‐based model at older ages for both sexes. MA‐based demographic coefficients for the CND population (panel 2b) are consistently higher than TM‐based coefficients.

MA‐based coefficients for influential HCCs (panels 2c and 2d) show some striking decreases from the TM‐based coefficients. Differences in recorded prevalence between TM and MA populations seen in Exhibit A‐5 suggest that coding intensity drives some of these decreases. In particular, HCC280 (Chronic Obstructive Pulmonary Disease), HCC226 (Heart Failure), HCC37/HCC38 (Diabetes, where the coefficients are constrained to be equal), and HCC48 (Morbid Obesity) have much higher MA prevalence and much lower MA‐based coefficients. The dramatically lower MA‐based coefficient for HCC93 (RA) suggests that providers in MA plans may be adopting cost‐reducing biosimilar therapies at a faster pace than TM providers [15].

Equity concerns motivate a review of how changing from TM‐based to MA‐based models would impact population subgroups. Exhibit A‐6 summarizes predictive ratios (expected resource use over actual resource use) by sex, race and rurality. The higher the predictive ratio (PR), the greater an MA plan's incentive to enroll that subgroup. When TM‐based coefficients are applied to the MA population, we see a small incentive to attract female enrollees relative to males (relative PR = 102%). This incentive disappears when using MA‐based coefficients. TM‐based coefficients create an incentive to enroll non‐Hispanic Blacks relative to non‐Hispanic Whites [NHW] (relative PR = 104%); the relative PR diminishes to 102% using MA‐based coefficients. The TM‐based coefficients create large incentives to attract Hispanics (relative PR = 137%) and non‐Hispanic Asian/Pacific Islanders (relative PR = 139%) relative to NHW beneficiaries. These incentives are moderated but persist when MA‐based coefficients are used (relative PR = 128% [Hispanic] and 138% [non‐Hispanic Asian/Pacific Islanders]). A small disincentive to enroll rural relative to urban participants (relative PR = 98%) using TM‐based coefficients is moderated to 99% when MA‐based coefficients are used.

### 3.4 Robustness Checks

We compare the distribution of TM‐based HCC scores computed using allowed amounts rather than standardized fees in Exhibit A‐7. Use of allowed amounts increase the mean HCC score for the CNA and CND populations by 1.0%, with increases at lower percentiles balanced by decreases at the 50th—95th percentiles. This suggests that our fee schedule algorithm may be missing structural adjustments, impacting the measurement of resource use in a manner that is correlated with overall health morbidity. Geographic pricing differences in allowed amounts, smoothed out in our standardized fees, may be a reason for these structural differences. Because some conditions vary geographically (e.g., the “diabetes belt” across the southern states [19]), and provider reimbursements vary geographically, there may be correlations between the allowed amount measure of resource use and health risk.

We also replicated the TM‐ and MA‐based models using CMS's v22 software. The results parallel what we found using v28, but the deviations between scores from replicated and CMS coefficients are greater (Exhibit A‐8). Rather than the 1.7% decrease from CMS to TM‐based mean scores in the v28 comparison, we found a 9.8% decrease in the v22 comparison. Because the CMS v22 data source is 5 years older than for v28 data, it is not surprising that the re‐estimation adjustment is larger. The v22 TM‐based to MA‐based mean difference in HCC scores is −13.2% (Exhibit A‐9, coefficients in Exhibit A‐10), reduced to −8.9% in the v28 comparison. This suggests that CMS's goal of reducing sensitivity to coding intensity was at least partially met with the issuance of v28.

Using MA encounter data to develop risk models may be sensitive to diagnostic coding intensity. We use CRR/HRA encounters as proxies for coding intensity, estimating models with and without these records [6]. Because financial reimbursement was not historically linked to *submission* of encounter data to CMS, some MA contracts did not submit encounters for all patient care received. Thus, we also estimate models with and without enrollees in contracts submitting incomplete encounter data [16]. Exhibit A‐11 shows the distributions of the scores based on the four possible combinations of diagnosis and contract selection. Coefficients are all applied to the same MA data—with HCC indicators identified using all diagnoses and including enrollees in all contracts. Dropping CRR/HRA diagnoses when estimating coefficients causes a 5.7% increase in the mean HCC score. Removing enrollees in contracts with incomplete data submission when estimating the model caused an approximate 1% increase in mean HCC scores. We expect this 1% difference to decline in the future due to CMS's shift to using encounter data for risk adjustment rather than the Risk Adjustment Processing System (change fully phased in by 2022) [20], which provides an incentive for plans to maximize reporting of encounters. This is true even for capitated physician groups, making it less likely that recent MA utilization is undercounted.

In Exhibit A‐11, we apply coefficients developed without CRR/HRA diagnoses to an MA population whose HCC indicators are based on all diagnoses, resulting in a mean increase of 5.7% relative to our baseline MA results. However, if we exclude the CRR/HRA diagnoses both when estimating the model and when applying the coefficients to compute HCC scores, the 5.7% increase is moderated to a 0.5% increase. This identifies the importance of data consistency between model estimation and model application.

Excluding the COVID years (2020–2022 resource use matched to 2019–2021 lagged diagnoses) from model estimation had a greater impact in the MA‐based model than the TM‐based model (Exhibit A‐12). Eliminating the COVID years caused a 1.1% increase in 2022 TM HCC scores. In contrast, eliminating COVID years generated a 5.5% increase in mean HCC scores in the 2022 MA population. In both populations, the impact was greater at the higher percentiles. The MA/TM difference in influence of the COVID years is interesting because the recording of diagnoses in TM dropped proportionately more than in MA in 2020 and rebounded more slowly (Exhibit A‐5) – particularly for diabetes, dementia and morbid obesity, which are frequently listed as secondary diagnoses. We expect that reduced recording of marginal diagnoses would inflate TM coefficients more than MA coefficients. The fact that MA coefficients had greater inflation suggests that TM resource use also dropped during the pandemic, moderating the increase in TM coefficients.

## 4 Discussion

Important shifts in patterns of care in TM claims have occurred, even in the few years since CMS HCC v28 was developed. These changes are expected, given evolution in medical technology, shifts in public health, trends in coding practices and health record systems, and advances in care management. For example, recent growth in the use of immunotherapy and precision medicine for cancer treatment cancer [21] may be driving the increased coefficients for HCC17 and HCC18, and the emergence of lower‐cost biosimilars could be driving savings in RA treatment (HCC93) [22, 23], as seen in Figure 1. In addition, strong trends in recorded prevalence are apparent in the 6 years shown in Exhibit A‐5, particularly for morbid obesity (HCC48). These trends could affect the associations between recorded diagnoses and expected resource use. CMS recognized the need to use more recent data in their 2027 Advanced Notice, in which they proposed to update v28 (implemented beginning in 2024) from the original basis of 2018 diagnoses to 2023 diagnoses [24].

Our MA‐based model resulted in lower health risk scores than the TM‐based model when both are applied to the MA population. The difference in coefficients (Figure 2) driving this difference in HCC scores is correlated with differences in TM and MA prevalence (Exhibit A‐5). Consider the large coefficient difference for Morbid Obesity (HCC48), where MA prevalence is 88% higher than TM prevalence, in contrast to the nearly identical coefficients for Specified Heart Arrhythmias (HCC238), where the difference in TM and MA prevalence is < 5%. The difference in the RA coefficient (HCC93), where lower‐cost biosimilars offer an alternative to biologics, suggests that MA plans adopt these cost‐saving therapies faster. However, this raises the policy question—should MA plans be penalized by reduced coefficients for this accelerated adoption?

While the MA‐based models lowered HCC scores for the MA population, we found that the new coefficients did not create new disincentives for MA plans to enroll beneficiaries who are ethnic or racial minorities, and a small disincentive to enroll rural beneficiaries was moderated under MA‐based coefficients. Thus, new equity concerns were not raised by the shift to MA‐based coefficients.

The use of standardized fees rather than TM allowed amounts or MA proprietary payment amounts has policy implications. Geographic price adjustment is the primary factor in CMS payment that is smoothed out by our standardized fees, which is a likely source of the differences we found across the HCC score distribution in Exhibit A‐7. Analogously, provider reimbursement differences due to MA plan negotiating power or capitation of segments of care would be smoothed out by replacing MA provider payments with standardized fees. Because the standardized fees provide a price‐neutral measure of resource use, this may be a preferred basis for developing risk adjustment models.

There are some limitations to our work. We use the CMS v28 model structure for the MA population without attempting to judge the appropriateness of that structure in the context of MA care delivery. We leave recalibration of model structure and exploration of non‐linear and machine learning estimation to future research. Our approach also does not allow us to separate the impact of differences due to favorable actions, such as care coordination and adoption of cost‐saving technologies, from differences due to coding intensity, though our examination of individual coefficients suggests that both differences are present.

The 20% sample in our six‐year cohort was chosen to increase sample size used to estimate the CMS v28 model, and balance COVID experience with pre‐COVID years. As demonstrated in Exhibit A‐5, some conditions had significant changes in prevalence over 6 years. The shorter timeframe of a 100% sample would produce coefficients that are more contemporary with the anticipated use period. Our longer timeframe also increases the chance of under‐reporting MA encounters, which declines as the use of encounters for HCC calculations is phased in through 2022.

This work focuses on CNA and CND models. In addition to these groups, risk adjustment covers the dual‐eligible, institutionalized, and new‐entrant populations. Evaluation of using encounter data for risk adjustment for all these groups needs to be conducted.

We demonstrate that MA‐based risk adjustment leads to meaningful differences in health risk scores, compared to TM‐based risk adjustment. However, care should be taken in using MA‐based risk adjusters grafted onto a payment system that uses baseline reimbursement from the TM sector [13, 14]. Finally, our MA‐based risk models reflect *average* coding intensity in MA. Between‐plan differences in coding intensity may remain [9, 25].

Our work provides an important contribution informing the policy debate about MA risk adjustment. We provide evidence, in both TM‐ and MA‐based models, that elevated recording of diagnoses leads to lower HCC coefficients as the marginal beneficiaries included in a coefficient's estimation are healthier. Our findings also suggest that MA plans may be adopting cost‐saving technologies, particularly for RA treatment, at a faster pace than TM providers, reducing relevant coefficients. On average, MA‐based coefficients provide substantially lower HCC scores than TM‐based coefficients, with the reduction peaking at the 50th and 75th percentiles, but subgroup analysis suggests that no additional disincentives to enroll racial or ethnic minorities (vs. NHW) or rural (vs. urban) beneficiaries result. Additional research is needed to understand the source of variation in the impact of moving from allowed amounts to standardized fees across the HCC distribution.

## Funding

This work was supported by the National Institute on Aging (1R01AG069352‐01A1). The content is solely the responsibility of the authors and does not necessarily represent the official views of the NIA.

## Conflicts of Interest

The authors declare no conflicts of interest.

## Supporting information


**Exhibit A‐1.** Hierarchies with constrained coefficients.
**Exhibit A‐2**. Characteristics of combined community‐dwelling, non‐dual, aged and disabled beneficiary/enrollee sample and their neighborhoods.
**Exhibit A‐3**. Characteristics of community‐dwelling, non‐dual, aged beneficiary/enrollee sample and their neighborhoods.
**Exhibit A‐4**. Characteristics of community‐dwelling, non‐dual, disabled beneficiary/enrollee sample and their neighborhoods.
**Exhibit A‐5**. Trends in prevalence of ten most influential HCCs.
**Exhibit A‐6**. 2022 predictive ratios by demographic groups.
**Exhibit A‐7**. Distribution of v28 HCC scores from TM‐based coefficients based on standardized fees and applied amounts, applied to the TM population.
**Exhibit A‐8**. Distribution of v22 HCC scores from CMS and TM‐based coefficients, applied to the TM population.
**Exhibit A‐9**. Distribution of v22 HCC scores from TM‐based and MA‐based coefficients, applied to the MA population.
**Exhibit A‐10**. TM‐ and MA‐based demographic and influential HCC coefficients from v22 model structure.
**Exhibit A‐11**. Distribution of v28 HCC scores from MA‐based coefficients by scope of data, applied to MA population.
**Exhibit A‐12**. Sample distribution of v28 HCC scores for models with and without COVID experience.

## Data Availability

The data that support the findings of this study are available from Centers for Medicare and Medicaid Services. Restrictions apply to the availability of these data, which were used under a data use agreement for this study. Data access can be requested from the Centers for Medicare and Medicaid Services at DataRequests@cms.hhs.gov.
