# Supplementary material for: Risk Adjustment in the Medicare Advantage Population Using Encounter Data
Source: Health Serv Res. 2026 Sep 10;61(5):e70165. doi: 10.1111/1475-6773.70165 (PMC13560787; doi:10.1111/1475-6773.70165)
Supplement: Supplementary file 1 [file HESR-61-e70165-s001.docx]

Supplementary Materials for

Risk Adjustment in the Medicare Advantage Population

Using Encounter Data

Caroline S. Carlin, PhD ^a^

Roger Feldman, PhD ^b^

Jeah Jung, PhD ^c^

July 30, 2026

^a^ Department of Family Medicine and Community Health, School of Medicine, University of Minnesota, Minneapolis, MN, USA. Email: [ccarlin@umn.edu](mailto:ccarlin@umn.edu)

^b^ Division of Health Policy and Management, School of Public Health, University of Minnesota, Minneapolis, MN, USA. Email: [feldm002@umn.edu](mailto:%20feldm002@umn.edu)

^c^ Department of Health Administration and Policy, College of Public Health, George Mason University, Fairfax, VA, USA. Email: [jjung41@gmu.edu](mailto:jjung41@gmu.edu)

This study was supported by the National Institute on Aging (NIA) 1R01AG069352-01A1. The content is solely the responsibility of the authors and does not necessarily represent the official views of the NIA.

Contents

[Exhibit A-1 – Hierarchies with Constrained Coefficients	3](#_Toc235679714)

[Exhibit A-2 – Characteristics of Combined Community-Dwelling, Non-dual, Aged and Disabled Beneficiary/Enrollee Sample and Their Neighborhoods	4](#_Toc235679715)

[Exhibit A-3 – Characteristics of Community-Dwelling, Non-dual, Aged Beneficiary/Enrollee Sample and Their Neighborhoods	5](#_Toc235679716)

[Exhibit A-4 – Characteristics of Community-Dwelling, Non-dual, Disabled Beneficiary/Enrollee Sample and Their Neighborhoods	6](#_Toc235679717)

[Exhibit A-5 – Trends in Prevalence of Ten Most Influential HCCs	7](#_Toc235679718)

[Exhibit A-6 – 2022 Predictive Ratios by Demographic Groups	10](#_Toc235679719)

[Exhibit A-7 – Distribution of v28 HCC Scores from TM-Based Coefficients Based on Standardized Fees and Applied Amounts, Applied to the TM Population	11](#_Toc235679720)

[Exhibit A-8 – Distribution of v22 HCC Scores from CMS and TM-Based Coefficients, Applied to the TM Population	12](#_Toc235679721)

[Exhibit A-9 – Distribution of v22 HCC Scores from TM-Based and MA-Based Coefficients, Applied to the MA Population	13](#_Toc235679722)

[Exhibit A-10 – TM- and MA-Based Demographic and Influential HCC Coefficients from v22 Model Structure	14](#_Toc235679723)

[Exhibit A-11 – Distribution of v28 HCC Scores from MA-Based Coefficients by Scope of Data, Applied to MA Population	16](#_Toc235679724)

[Exhibit A-12 - Sample Distribution of v28 HCC Scores for Models with and without COVID Experience	18](#_Toc235679725)

# Exhibit A-1 – Hierarchies with Constrained Coefficients

| ***Version 28 Hierarchies with Equal Coefficients across HCCs*** | |
| --- | --- |
| HCC36 | Diabetes with Severe Acute Complications |
| HCC37 | Diabetes with Chronic Complications |
| HCC38 | Diabetes with Glycemic, Unspecified, or No Complications |
| HCC125 | Dementia, Severe |
| HCC126 | Dementia, Moderate |
| HCC127 | Dementia, Mild or Unspecified |
| HCC135 | Drug Use with Psychotic Complications |
| HCC136 | Alcohol Use with Psychotic Complications |
| HCC212 | Respiratory Arrest |
| HCC213 | Cardio-Respiratory Failure and Shock |
| HCC222 | End-Stage Heart Failure |
| HCC223 | Heart Failure with Heart Assist Device/Artificial Heart |
| HCC224 | Acute on Chronic Heart Failure |
| HCC225 | Acute Heart Failure (Excludes Acute on Chronic) |
| HCC226 | Heart Failure, Except End-Stage and Acute |
| HCC248 | Intracranial Hemorrhage |
| HCC249 | Ischemic or Unspecified Stroke |
| HCC328 | Chronic Kidney Disease, Moderate (Stage 3B) |
| HCC329 | Chronic Kidney Disease, Moderate (Stage 3, Except 3B) |
| HCC397 | Major Head Injury with Loss of Consciousness > 1 Hour |
| HCC398 | Major Head Injury with Loss of Consciousness < 1 Hour or Unspecified |
| HCC399 | Major Head Injury without Loss of Consciousness |
| ***Version 22 Hierarchies with Equal Coefficients across HCCs*** | |
| HCC17 | Diabetes with Acute Complications |
| HCC18 | Diabetes with Chronic Complications |
| HCC134 | Dialysis Status |
| HCC135 | Acute Renal Failure |
| HCC136 | Chronic Kidney Disease, Stage 5 |
| HCC137 | Chronic Kidney Disease, Severe (Stage 4) |

Abbreviations: HCC = Hierarchical Condition Category

# Exhibit A-2 – Characteristics of Combined Community-Dwelling, Non-dual, Aged and Disabled Beneficiary/Enrollee Sample and Their Neighborhoods

|  | Traditional Medicare | Medicare Advantage | p-value |
| --- | --- | --- | --- |
| Beneficiary-years (n [%]) | 29,884,452 [100%] | 22,100,138 [100%] |  |
| Aged | 27,975,229 [94%] | 20,503,541 [93%] |  |
| Disabled | 1,909,223 [6%] | 1,596,597 [7%] |  |
| Demographics |  |  |  |
| Age (mean [SD]) | 73.4 [9.2] | 73.2 [8.5] | <.0001 |
| Female (%) | 54.0% | 54.8% | <.0001 |
| Race (%) |  |  |  |
| White, non-Hispanic | 86.3% | 75.1% | <.0001 |
| Black, non-Hispanic | 6.4% | 9.6% |  |
| Asian, non-Hispanic | 2.0% | 3.2% |  |
| Other race, non-Hispanic | 3.1% | 2.6% |  |
| Hispanic | 4.0% | 11.0% |  |
| Geographic descriptors |  |  |  |
| American Community Survey (mean [SD]) |  |  |  |
| Percent population age 25+ in ZIP with 4-year degree or higher | 33.4 [17.3] | 31.8 [16.1] | <.0001 |
| Percent population age 5+ in ZIP speaking English only | 87.3 [15.0] | 85.3 [18.3] | <.0001 |
| Percent households in ZIP under the Federal Poverty Limit | 11.9 [7.4] | 12.5 [7.9] | <.0001 |
| Median household income in ZIP | 72,397 [30,144] | 70,418 [27,646] | <.0001 |
| Area Health Resource Files (mean [SD]) |  |  |  |
| Hospital beds per 1000 population in county | 2.9 [2.3] | 2.9 [2.0] | <.0001 |
| MDs per 1000 population in county | 3.3 [2.5] | 3.4 [2.3] | <.0001 |
| SNF beds per 1000 population in county | 5.7 [3.3] | 5.1 [2.9] | <.0001 |
| Rural (%) | 26.0% | 17.8% | <.0001 |
| Health descriptors |  |  |  |
| CMS v28 HCC score (mean [SD]) | 0.96 [0.94] | 1.01 [0.95] | <.0001 |
| Fee-based measure of monthly resource use (mean [SD]) | $1,058 [2,977] | $839 [3,171] | <.0001 |

Abbreviations: CMS HCC = Centers for Medicare and Medicaid Services Hierarchical Condition Category, version 28

# Exhibit A-3 – Characteristics of Community-Dwelling, Non-dual, Aged Beneficiary/Enrollee Sample and Their Neighborhoods

|  | Traditional Medicare | Medicare Advantage | p-value |
| --- | --- | --- | --- |
| Beneficiary-years (n) | 27,975,229 | 20,503,541 |  |
| Demographics |  |  |  |
| Age (mean [SD]) | 74.8 [7.6] | 74.5 [7.2] | <.0001 |
| Female (%) | 54.7% | 55.2% | <.0001 |
| Race (%) |  |  |  |
| White, non-Hispanic | 87.2% | 75.8% | <.0001 |
| Black, non-Hispanic | 5.9% | 9.3% |  |
| Asian, non-Hispanic | 2.0% | 3.3% |  |
| Other race, non-Hispanic | 3.2% | 2.6% |  |
| Hispanic | 3.7% | 10.5% |  |
| Geographic descriptors |  |  |  |
| American Community Survey (mean [SD]) |  |  |  |
| Percent population age 25+ in ZIP with 4-year degree or higher | 33.8 [17.4] | 32.2 [16.2] | <.0001 |
| Percent population age 5+ in ZIP speaking English only | 87.3 [14.9] | 85.2 [18.3] | <.0001 |
| Percent households in ZIP under the Federal Poverty Limit | 11.7 [7.3] | 12.4 [7.8] | <.0001 |
| Median household income in ZIP | 72,997 [30,376] | 71,065 [27,840] | <.0001 |
| Area Health Resource Files (mean [SD]) |  |  |  |
| Hospital beds per 1000 population in county | 2.9 [2.3] | 2.9 [2.0] | <.0001 |
| MDs per 1000 population in county | 3.3 [2.5] | 3.4 [2.3] | <.0001 |
| SNF beds per 1000 population in county | 5.7 [3.3] | 5.1 [2.9] | <.0001 |
| Rural (%) | 25.8% | 17.4% | <.0001 |
| Health descriptors |  |  |  |
| CMS v28 HCC score (mean [SD]) | 0.96 [0.92] | 1.00 [0.92] | <.0001 |
| Fee-based measure of monthly resource use (mean [SD]) | $1,060 [2,953] | $828 [3,142] | <.0001 |

Abbreviations: CMS HCC = Centers for Medicare and Medicaid Services Hierarchical Condition Category, version 28

# Exhibit A-4 – Characteristics of Community-Dwelling, Non-dual, Disabled Beneficiary/Enrollee Sample and Their Neighborhoods

|  | Traditional Medicare | Medicare Advantage | p-value |
| --- | --- | --- | --- |
| Beneficiary-years (n) | 1,909,223 | 1,596,597 |  |
| Demographics |  |  |  |
| Age (mean [SD]) | 53.9 [8.8] | 56.3 [6.9] | <.0001 |
| Female (%) | 44.5% | 48.8% | <.0001 |
| Race (%) |  |  |  |
| White, non-Hispanic | 74.2% | 67.0% | <.0001 |
| Black, non-Hispanic | 14.8% | 14.1% |  |
| Asian, non-Hispanic | 1.7% | 1.3% |  |
| Other race, non-Hispanic | 2.7% | 1.6% |  |
| Hispanic | 7.8% | 16.7% |  |
| Geographic descriptors |  |  |  |
| American Community Survey (mean [SD]) |  |  |  |
| Percent population age 25+ in ZIP with 4-year degree or higher | 27.3 [14.6] | 26.3 [13.8] | <.0001 |
| Percent population age 5+ in ZIP speaking English only | 87.0 [15.9] | 86.6 [17.4] | <.0001 |
| Percent households in ZIP under the Federal Poverty Limit | 14.0 [8.2] | 14.8 [8.7] | <.0001 |
| Median household income in ZIP | 63,540 [24,864] | 61,673 [23,163] | <.0001 |
| Area Health Resource Files (mean [SD]) |  |  |  |
| Hospital beds per 1000 population in county | 3.0 [2.4] | 2.9 [2.2] | <.0001 |
| MDs per 1000 population in county | 3.0 [2.3] | 3.0 [2.2] | <.0001 |
| SNF beds per 1000 population in county | 5.8 [3.3] | 5.1 [3.3] | <.0001 |
| Rural (%) | 28.9% | 23.5% | <.0001 |
| Health descriptors |  |  |  |
| CMS v28 HCC score (mean [SD]) | 0.91 [1.18] | 1.11 [1.21] | <.0001 |
| Fee-based measure of monthly resource use (mean [SD]) | $1,037 [3,310] | $984 [3,527] | <.0001 |

Abbreviations: CMS HCC = Centers for Medicare and Medicaid Services Hierarchical Condition Category, version 28

# Exhibit A-5 – Trends in Prevalence of Ten Most Influential HCCs

| ***HCC280: Chronic Obstructive Pulmonary Disease, Interstitial Lung Disorders, and Other Chronic Lung Disorders*** | | | |
| --- | --- | --- | --- |
|  | TM | MA | MA difference from TM |
| 2016 | 0.108 | 0.140 | 0.031 [28.9%] |
| 2017 | 0.109 | 0.143 | 0.035 [31.9%] |
| 2018 | 0.108 | 0.146 | 0.038 [35.7%] |
| 2019 | 0.107 | 0.148 | 0.041 [38.1%] |
| 2020 | 0.097 | 0.140 | 0.043 [45.0%] |
| 2021 | 0.097 | 0.143 | 0.046 [46.9%] |
| Growth 2016 to 2021 | -0.011 [-10.2%] | 0.003 [2.3%] |  |
| ***HCC226: Heart Failure, Except End-Stage and Acute*** | | | |
|  | TM | MA | MA difference from TM |
| 2016 | 0.060 | 0.077 | 0.016 [26.6%] |
| 2017 | 0.064 | 0.083 | 0.019 [30.0%] |
| 2018 | 0.071 | 0.096 | 0.024 [34.2%] |
| 2019 | 0.074 | 0.100 | 0.025 [34.2%] |
| 2020 | 0.071 | 0.098 | 0.027 [38.3%] |
| 2021 | 0.075 | 0.103 | 0.028 [37.3%] |
| Growth 2016 to 2021 | 0.014 [23.6%] | 0.026 [34.0%] |  |
| ***HCC238: Specified Heart Arrhythmias*** | | | |
|  | TM | MA | MA difference from TM |
| 2016 | 0.117 | 0.110 | -0.008 [-6.8%] |
| 2017 | 0.119 | 0.112 | -0.007 [-5.7%] |
| 2018 | 0.121 | 0.115 | -0.006 [-5.0%] |
| 2019 | 0.128 | 0.120 | -0.008 [-6.0%] |
| 2020 | 0.127 | 0.120 | -0.007 [-5.6%] |
| 2021 | 0.133 | 0.124 | -0.009 [-6.7%] |
| Growth 2016 to 2021 | 0.015 [13.1%] | 0.015 [13.2%] |  |
| ***HCC037: Diabetes with Chronic Complications*** | | | |
|  | TM | MA | MA difference from TM |
| 2016 | 0.094 | 0.161 | 0.067 [71.4%] |
| 2017 | 0.106 | 0.178 | 0.072 [67.4%] |
| 2018 | 0.111 | 0.187 | 0.076 [68.0%] |
| 2019 | 0.116 | 0.193 | 0.077 [66.2%] |
| 2020 | 0.112 | 0.188 | 0.076 [68.0%] |
| 2021 | 0.118 | 0.197 | 0.079 [67.2%] |
| Growth 2016 to 2021 | 0.023 [24.8%] | 0.035 [21.8%] |  |

| ***HCC017: Cancer Metastatic to Lung, Liver, Brain, and Other Organs; Acute Myeloid Leukemia Except Promyelocytic*** | | | |
| --- | --- | --- | --- |
|  | TM | MA | MA difference from TM |
| 2016 | 0.0068 | 0.0055 | -0.0012 [-18.3%] |
| 2017 | 0.0070 | 0.0059 | -0.0011 [-16.3%] |
| 2018 | 0.0073 | 0.0061 | -0.0012 [-15.9%] |
| 2019 | 0.0077 | 0.0064 | -0.0012 [-16.3%] |
| 2020 | 0.0075 | 0.0062 | -0.0013 [-17.2%] |
| 2021 | 0.0080 | 0.0066 | -0.0015 [-18.2%] |
| Growth 2016 to 2021 | 0.0012 [18.3%] | 0.0010 [18.6%] |  |
| ***HCC093: Rheumatoid Arthritis and Other Specified Inflammatory Rheumatic Disorders*** | | | |
|  | TM | MA | MA difference from TM |
| 2016 | 0.029 | 0.032 | 0.003 [10.3%] |
| 2017 | 0.029 | 0.033 | 0.004 [13.1%] |
| 2018 | 0.030 | 0.035 | 0.005 [15.1%] |
| 2019 | 0.031 | 0.036 | 0.004 [14.1%] |
| 2020 | 0.030 | 0.035 | 0.005 [15.4%] |
| 2021 | 0.032 | 0.037 | 0.005 [15.1%] |
| Growth 2016 to 2021 | 0.003 [12.1%] | 0.005 [16.9%] |  |
| ***HCC048: Morbid Obesity*** | | | |
|  | TM | MA | MA difference from TM |
| 2016 | 0.040 | 0.080 | 0.041 [102.2%] |
| 2017 | 0.045 | 0.088 | 0.043 [94.8%] |
| 2018 | 0.051 | 0.095 | 0.044 [86.7%] |
| 2019 | 0.056 | 0.102 | 0.046 [81.5%] |
| 2020 | 0.054 | 0.100 | 0.046 [86.6%] |
| 2021 | 0.061 | 0.110 | 0.049 [80.8%] |
| Growth 2016 to 2021 | 0.021 [53.0%] | 0.030 [36.8%] |  |
| ***HCC127: Dementia, Mild or Unspecified*** | | | |
|  | TM | MA | MA difference from TM |
| 2016 | 0.046 | 0.045 | -0.001 [-1.7%] |
| 2017 | 0.046 | 0.046 | 0.001 [1.5%] |
| 2018 | 0.045 | 0.047 | 0.002 [4.0%] |
| 2019 | 0.045 | 0.049 | 0.004 [9.0%] |
| 2020 | 0.040 | 0.046 | 0.006 [13.8%] |
| 2021 | 0.042 | 0.048 | 0.006 [14.4%] |
| Growth 2016 to 2021 | -0.004 [-8.3%] | 0.003 [6.7%] |  |

| ***HCC018: Cancer Metastatic to Bone, Other and Unspecified Metastatic Cancer; Acute Leukemia Except Myeloid*** | | | | | | |
| --- | --- | --- | --- | --- | --- | --- |
|  | TM | | MA | | | MA difference from TM |
| 2016 | 0.0055 | | 0.0051 | | | -0.0004 [-8.1%] |
| 2017 | 0.0058 | | 0.0055 | | | -0.0003 [-5.3%] |
| 2018 | 0.0061 | | 0.0058 | | | -0.0003 [-5.0%] |
| 2019 | 0.0064 | | 0.0060 | | | -0.0004 [-5.9%] |
| 2020 | 0.0064 | | 0.0059 | | | -0.0005 [-7.2%] |
| 2021 | 0.0068 | | 0.0062 | | | -0.0006 [-8.2%] |
| Growth 2016 to 2021 | 0.0013 [23.0%] | | 0.0012 [22.8%] | | |  |
| ***HCC038: Diabetes with Glycemic, Unspecified, or No Complications*** | | | | | | |
|  | TM | | MA | | | MA difference from TM |
| 2016 | 0.133 | | 0.119 | | | -0.015 [-11.1%] |
| 2017 | 0.120 | | 0.104 | | | -0.016 [-13.6%] |
| 2018 | 0.114 | | 0.095 | | | -0.019 [-16.7%] |
| 2019 | 0.109 | | 0.089 | | | -0.020 [-18.1%] |
| 2020 | 0.103 | | 0.087 | | | -0.016 [-15.6%] |
| 2021 | 0.101 | | 0.084 | | | -0.017 [-16.6%] |
| Growth 2016 to 2021 | -0.033 [-24.4%] | | -0.035 [-29.1%] | | |  |
| ***HCC037 and HCC038: Diabetes with Chronic Complications and Diabetes with Glycemic, Unspecified, or No Complications*** | | | | | | |
|  | | TM | | MA | MA difference from TM | |
| 2016 | | 0.228 | | 0.280 | 0.052 [23.1%] | |
| 2017 | | 0.227 | | 0.282 | 0.055 [24.5%] | |
| 2018 | | 0.225 | | 0.282 | 0.057 [25.2%] | |
| 2019 | | 0.225 | | 0.282 | 0.057 [25.4%] | |
| 2020 | | 0.215 | | 0.275 | 0.060 [27.9%] | |
| 2021 | | 0.218 | | 0.281 | 0.062 [28.5%] | |
| Growth 2016 to 2021 | | -0.009 [-4.0%] | | 0.001 [0.2%] |  | |

Notes: Prevalence computed using combined Community-dwelling, Non-dual Aged (CNA) and Community-dwelling, Non-dual Disabled (CND) populations. HCCs are considered “influential” if their mean contribution to the HCC score (through high prevalence and/or acuity) ranks in the top 10 contributions across the 115 v28 HCCs in the CNA and CND models, based on the published CMS v28 coefficients. On average, these 10 HCCs make up 25.1% of the total CMS v28 HCC scores in the MA CNA and CND populations.

Abbreviations: HCC=Hierarchical Condition Category; MA=Medicare Advantage; TM=Traditional Medicare

# Exhibit A-6 – 2022 Predictive Ratios by Demographic Groups

|  | TM-Based Model, Applied to TM Population | | TM-Based Model, Applied to MA Population | | MA-Based Model, Applied to MA Population | |
| --- | --- | --- | --- | --- | --- | --- |
|  | Predictive Ratio | Relative Predictive Ratio (Slope) | Predictive Ratio | Relative Predictive Ratio (Slope) | Predictive Ratio | Relative Predictive Ratio (Slope) |
| Total Population | 1.01 |  | 1.14 |  | 1.04 |  |
| By Sex |  |  |  |  |  |  |
| Male | 1.02 | 1.00 | 1.13 | 1.00 | 1.05 | 1.00 |
| Female | 1.02 | 1.00 | 1.15 | 1.02 | 1.04 | 0.99 |
| By Race |  |  |  |  |  |  |
| Non-Hispanic White | 1.00 | 1.00 | 1.10 | 1.00 | 1.01 | 1.00 |
| Non-Hispanic Black | 1.09 | 1.09 | 1.14 | 1.04 | 1.03 | 1.02 |
| Hispanic | 1.16 | 1.16 | 1.50 | 1.37 | 1.30 | 1.28 |
| Non-Hispanic Asian/ Pacific Islander | 1.25 | 1.25 | 1.53 | 1.39 | 1.40 | 1.38 |
| Other | 1.05 | 1.05 | 1.20 | 1.09 | 1.10 | 1.09 |
| By Rurality |  |  |  |  |  |  |
| Urban | 1.00 | 1.00 | 1.13 | 1.00 | 1.03 | 1.00 |
| Rural | 1.03 | 1.03 | 1.11 | 0.98 | 1.02 | 0.99 |

Notes:

1. Predictive Ratios are computed as (expected resource use)/(actual resource use) using the 2022 TM and MA CNA/CND sample. The average predictive ratio across the 2022 sample is slightly higher than the expected 1.00 in the first and third sets of predictive ratios, because average prevalences trend higher for 2022 beneficiaries than the average prevalences over the 2017-2022 period used to estimate coefficients.
2. TM-Based model based on coefficients estimated from TM data using standardized fees to compute relative resource use, applied to 2022 TM or MA CNA and CND beneficiaries.
3. MA-Based model based on coefficients estimated from MA data using standardized fees to compute relative resource use, applied to 2022 MA CNA and CND beneficiaries.

Abbreviations: CNA=community-dwelling, non-dual aged population; CND=community-dwelling, non-dual disabled population; MA=Medicare Advantage; TM=Traditional Medicare

# Exhibit A-7 – Distribution of v28 HCC Scores from TM-Based Coefficients Based on Standardized Fees and Applied Amounts, Applied to the TM Population

|  | Based on TM Standardized Fees | Based on TM Allowed Amounts | Difference |
| --- | --- | --- | --- |
| Mean | 0.961 | 0.971 | 0.010 [1.0%] |
| Percentiles |  |  |  |
| 1% | 0.284 | 0.305 | 0.021 [7.4%] |
| 5% | 0.321 | 0.323 | 0.002 [0.7%] |
| 10% | 0.340 | 0.349 | 0.009 [2.7%] |
| 25% | 0.393 | 0.395 | 0.001 [0.3%] |
| 50% | 0.599 | 0.595 | -0.003 [-0.6%] |
| 75% | 1.081 | 1.057 | -0.023 [-2.2%] |
| 90% | 1.975 | 1.937 | -0.037 [-1.9%] |
| 95% | 2.939 | 2.895 | -0.044 [-1.5%] |
| 99% | 5.620 | 5.681 | 0.060 [1.1%] |

1. First TM-based distribution based on coefficients estimated from TM data using standardized fees to compute relative resource use (baseline TM-based model), applied to TM CNA and CND beneficiaries.
2. Second TM-based distribution based on coefficients estimated from TM data using allowed amounts to compute relative resource use, applied to TM CNA and CND beneficiaries.

Abbreviations: CNA=community-dwelling, non-dual aged population; CND=community-dwelling, non-dual disabled population; HCC= Hierarchical Condition Category; TM=Traditional Medicare

# Exhibit A-8 – Distribution of v22 HCC Scores from CMS and TM-Based Coefficients, Applied to the TM Population

|  | Using CMS Coefficients | Using TM-Based Coefficients | Difference |
| --- | --- | --- | --- |
| Mean | 1.065 | 0.961 | -0.104 [-9.8%] |
| Percentiles |  |  |  |
| 1% | 0.300 | 0.289 | -0.011 [-3.6%] |
| 5% | 0.300 | 0.298 | -0.002 [-0.5%] |
| 10% | 0.312 | 0.316 | 0.004 [1.4%] |
| 25% | 0.416 | 0.370 | -0.046 [-11.1%] |
| 50% | 0.734 | 0.607 | -0.127 [-17.4%] |
| 75% | 1.296 | 1.090 | -0.206 [-15.9%] |
| 90% | 2.210 | 1.990 | -0.220 [-9.9%] |
| 95% | 3.082 | 2.969 | -0.113 [-3.7%] |
| 99% | 5.130 | 5.326 | 0.196 [3.8%] |

Notes:

3. CMS v22 distributions based on published coefficients in the CMS v22 software, applied to TM CNA and CND beneficiaries.
4. TM-based distribution based on coefficients estimated from TM data using standardized fees to compute relative resource use, applied to TM CNA and CND beneficiaries, using v22 model structure.

Abbreviations: CMS=Centers for Medicare and Medicaid Services; CNA=community-dwelling, non-dual aged population; CND=community-dwelling, non-dual disabled population; HCC=Hierarchical Condition Category; TM=Traditional Medicare

# Exhibit A-9 – Distribution of v22 HCC Scores from TM-Based and MA-Based Coefficients, Applied to the MA Population

|  | Using  TM-Based Coefficients | Using MA-Based Coefficients | Difference |
| --- | --- | --- | --- |
| Mean | 1.083 | 0.941 | -0.143 [-13.2%] |
| Percentiles |  |  |  |
| 1% | 0.298 | 0.304 | 0.006 [1.9%] |
| 5% | 0.298 | 0.304 | 0.006 [1.9%] |
| 10% | 0.316 | 0.331 | 0.015 [4.7%] |
| 25% | 0.418 | 0.406 | -0.012 [-2.9%] |
| 50% | 0.707 | 0.598 | -0.109 [-15.5%] |
| 75% | 1.290 | 1.032 | -0.258 [-20.0%] |
| 90% | 2.285 | 1.915 | -0.369 [-16.2%] |
| 95% | 3.256 | 2.854 | -0.402 [-12.3%] |
| 99% | 5.571 | 5.172 | -0.399 [-7.2%] |

 Notes:

1. TM-based distribution based on coefficients estimated from TM data using standardized fees to compute relative resource use, applied to MA CNA and CND enrollees, using v22 model structure.
2. MA-based distribution based on coefficients estimated from MA data using standardized fees to compute relative resource use, applied to MA CNA and CND enrollees, using v22 model structure.

Abbreviations: CNA=community-dwelling, non-dual aged population; CND=community-dwelling, non-dual disabled population; HCC=Hierarchical Condition Category; MA=Medicare Advantage; TM=Traditional Medicare

# Exhibit A-10 – TM- and MA-Based Demographic and Influential HCC Coefficients from v22 Model Structure

Notes:

1. TM-based coefficients are estimated from TM data using standardized fees to compute relative resource use and applied to the MA population.
2. MA-based coefficients are estimated from MA data using standardized fees to compute relative resource use.
3. HCC coefficients that are influential due to high prevalence vs. high acuity are shown separately to allow for differently proportioned y-axes.
4. Influential HCC v22 descriptors in descending order of contribution to mean HCC score, based on CMS published coefficients applied to combined TM/MA and CNA/CND populations: HCC18=Diabetes with Chronic Complications; HCC108=Vascular Disease; HCC58=Major Depressive Bipolar and Paranoid Disorders; HCC85=Congestive Heart Failure; HCC96=Specified Heart Arrhythmias; HCC111=Chronic Obstructive Pulmonary Disease; HCC8=Metastatic Cancer and Acute Leukemia; HCC40=Rheumatoid Arthritis and Inflammatory Connective Tissue Disease; HCC40=Rheumatoid Arthritis and Inflammatory Connective Tissue Disease; HCC22=Morbid Obesity; HCC48=Coagulation Defects and Other Specified Hematological Disorders

Abbreviations: CMS=Centers for Medicare and Medicaid Services; CNA=community-dwelling, non-dual aged population; CND=community-dwelling, non-dual disabled population; HCC=Hierarchical Condition Category; MA=Medicare Advantage; TM=Traditional Medicare

# Exhibit A-11 – Distribution of v28 HCC Scores from MA-Based Coefficients by Scope of Data, Applied to MA Population

|  | ***Including CRR/HRA*** | ***Excluding CRR/HRA*** | ***CRR/HRA Exclusion Difference*** |
| --- | --- | --- | --- |
| ***Enrollees in all contracts*** | | | |
| Mean | 0.941 | 0.994 | 0.053 [5.7%] |
| Percentiles |  |  |  |
| 1% | 0.315 | 0.320 | 0.005 [1.6%] |
| 5% | 0.315 | 0.320 | 0.005 [1.6%] |
| 10% | 0.344 | 0.349 | 0.005 [1.4%] |
| 25% | 0.398 | 0.412 | 0.014 [3.6%] |
| 50% | 0.590 | 0.630 | 0.040 [6.7%] |
| 75% | 1.037 | 1.100 | 0.064 [6.1%] |
| 90% | 1.904 | 2.031 | 0.127 [6.6%] |
| 95% | 2.771 | 2.948 | 0.177 [6.4%] |
| 99% | 5.419 | 5.699 | 0.280 [5.2%] |
| ***Enrollees in complete contracts*** | | | |
| Mean | 0.951 | 1.006 | 0.055 [5.7%] |
| Percentiles |  |  |  |
| 1% | 0.319 | 0.324 | 0.005 [1.6%] |
| 5% | 0.319 | 0.324 | 0.005 [1.6%] |
| 10% | 0.349 | 0.354 | 0.005 [1.4%] |
| 25% | 0.403 | 0.418 | 0.014 [3.6%] |
| 50% | 0.598 | 0.636 | 0.038 [6.4%] |
| 75% | 1.048 | 1.111 | 0.064 [6.1%] |
| 90% | 1.922 | 2.051 | 0.129 [6.7%] |
| 95% | 2.797 | 2.984 | 0.187 [6.7%] |
| 99% | 5.479 | 5.770 | 0.290 [5.3%] |
| ***Completeness Difference*** | | | |
| Mean | 0.010 [1.0%] | 0.011 [1.1%] |  |
| Percentiles |  |  |  |
| 1% | 0.005 [1.5%] | 0.005 [1.5%] |  |
| 5% | 0.005 [1.5%] | 0.005 [1.5%] |  |
| 10% | 0.005 [1.4%] | 0.005 [1.4%] |  |
| 25% | 0.005 [1.4%] | 0.006 [1.3%] |  |
| 50% | 0.008 [1.3%] | 0.006 [1.0%] |  |
| 75% | 0.011 [1.0%] | 0.011 [1.0%] |  |
| 90% | 0.017 [0.9%] | 0.020 [1.0%] |  |
| 95% | 0.026 [0.9%] | 0.036 [1.2%] |  |
| 99% | 0.060 [1.1%] | 0.071 [1.2%] |  |

Notes:

1. Distributions based on coefficients estimated from MA data using standardized fees to compute relative resource use, with a various subsets of data for coefficient estimation. Subsets are determined by whether enrollees in contracts with incomplete encounter submission are removed, and whether diagnosis appearing only in chart review records and health risk assessments are used to compute HCC indicators.
2. Once estimated, all four sets of MA-based coefficients are applied to the same independent variable dataset: CNA/CND MA enrollees regardless of contract completeness, with HCC indicators computed with all diagnosis, including those identified only on chart review records and health risk assessments.

Abbreviations: CNA=community-dwelling, non-dual aged population; CND= community-dwelling, non-dual disabled population; CRR=chart review record; HCC= Hierarchical Condition Category; HRA=health risk assessment; MA=Medicare Advantage

# Exhibit A-12 - Sample Distribution of v28 HCC Scores for Models with and without COVID Experience

|  | ***Baseline Model*** | ***Model Excluding COVID Years*** | ***Exclusion Difference*** |
| --- | --- | --- | --- |
| ***TM-Based Model*** | | | |
| Mean | 0.971 | 0.981 | 0.010 [1.1%] |
| Percentiles |  |  |  |
| 1% | 0.284 | 0.297 | 0.013 [4.5%] |
| 5% | 0.321 | 0.328 | 0.007 [2.1%] |
| 10% | 0.340 | 0.333 | -0.007 [-2.1%] |
| 25% | 0.393 | 0.398 | 0.005 [1.3%] |
| 50% | 0.599 | 0.610 | 0.011 [1.8%] |
| 75% | 1.081 | 1.084 | 0.003 [0.3%] |
| 90% | 1.975 | 2.016 | 0.041 [2.1%] |
| 95% | 2.939 | 3.004 | 0.065 [2.2%] |
| 99% | 5.620 | 5.647 | 0.027 [0.5%] |
| ***MA-Based Model*** | | | |
| Mean | 0.941 | 0.993 | 0.051 [5.5%] |
| Percentiles |  |  |  |
| 1% | 0.315 | 0.327 | 0.012 [3.9%] |
| 5% | 0.315 | 0.327 | 0.012 [3.9%] |
| 10% | 0.344 | 0.341 | -0.003 [-0.9%] |
| 25% | 0.398 | 0.416 | 0.018 [4.6%] |
| 50% | 0.590 | 0.625 | 0.034 [5.8%] |
| 75% | 1.037 | 1.077 | 0.040 [3.9%] |
| 90% | 1.904 | 2.067 | 0.163 [8.6%] |
| 95% | 2.771 | 2.998 | 0.227 [8.2%] |
| 99% | 5.419 | 5.676 | 0.257 [4.7%] |

Notes:

1. TM-based distribution based on coefficients estimated from TM data using standardized fees to compute relative resource use, applied to TM CNA and CND beneficiaries.
2. MA-based distribution based on coefficients estimated from MA data using standardized fees to compute relative resource use, applied to MA CNA and CND enrollees.
3. The COVID exclusion scenarios are based on coefficients estimated from data after excluding COVID impact, retaining 2016-2018 diagnoses predicting 2017-2019 utilization.

Abbreviations: CNA=community-dwelling, non-dual aged population; CND=community-dwelling, non-dual disabled population; HCC=Hierarchical Condition Category; MA=Medicare Advantage; TM=Traditional Medicare
